# Research and Post-Graduate Study in Medicine

**Published:** 1903-10-24

**Authors:** 


### The Hospital.

A Journal of

The Medical Sciences and Hospital Administration.

Vol. XXXV.—No. 891. OCTOBER 24, 1903.

### Research and Post-graduate Study in Medicine.

During the last few weeks the columns of the *Times* have borne ample testimony to the interest which is taken in research and post-graduate study in the Universities of Oxford and Cambridge. The bequest to the University of Oxford under the will of Mr. Cecil Rhodes has introduced a new order of students with different ideals who are not always willing to assimilate themselves to the existing conditions. There is already a cry for more advanced teaching and for more specialised instruction in various branches of knowledge. With this outcry the teachers in the medical profession have long been familiar and they have solved the difficulty with a very fair measure of success at home by the various post-graduate courses of lectures and demonstrations—in the United States by the foundation of such noble establishments as the Johns Hopkins University. The medical profession, however, is especially favoured in respect of research and post-graduate work. Everyone in medicine is obliged to undertake post-graduate study, for the work of his daily round compels him to advance his knowledge both by observation and by reading. It is not so in more academic studies. College tutors are heavily handicapped in original work and "their output is small, chiefly," as Mr. Warde Fowler says, "because there is, as a rule, no correlation between their own work and their teaching; in other words, what they are most deeply interested in is not what their pupils are interested in." There is no such complaint possible in the wards and out-patient rooms of our large hospitals where the crowds of students who sit round their physician or surgeon are quite as much interested as he is himself in any particular set of cases which he happens to be working out for the time being. Such teachers are peculiarly happily placed. The tutorial classes conducted solely with a view to examination requirements enable him to pick out the more promising students whilst it teaches him the places where knowledge is most wanted and thus shows where further work is needed. He is constantly asked for themes for the theses required for the higher degrees at the different universities, and this enables him to set the students to work upon fields which are likely to prove fertile. But the power of research is not given to every one, and the most brilliant men are not usually those who are able to undertake it. The most brilliant men are often the poorest, and it is usually a matter of the utmost importance that they should maintain themselves as soon as possible, and in the present state of science this cannot be done by research alone. In a large medical school, however, there are always a few individuals so fortunately situated as to be able to devote some portion of their time to unremunerative laboratory work. A skilful teacher, and one who is himself endowed with the gift for research will utilise these men to the uttermost. With their assistance many facts can be obtained to be skilfully woven afterwards into the fabric of the work. But much less use is made of such help in England than it is in Germany where every professor has several advanced students working under his supervision with the happiest results both to himself and his subordinates. The Germans therefore are noted for the accuracy of their detail and for the assiduity with which they hunt down every case which bears upon the subject on which they are engaged. Yet comparatively little is heard of the "Ghosts". In France, on the other hand, the pupil is much in evidence whilst the professor is relegated to the background. The candidate for the doctorate publishes an elaborate treatise which has been largely inspired by his teacher. Such theses are often superlatively good, and many, like our Jacksonian Prize essays, form the starting point of the writer's life work.

But if the power of original work is given only to a few, all alike receive benefit from post-graduate study, whether it is formally in class and by lectures or informally from the mere desire to keep abreast of the times. The advances in medical science are so great and so continuous that it is almost impossible to know them on every side. Whole branches of science have developed within the last twenty years, like bacteriology; new methods of cure have arisen like the light treatment; fresh means of diagnosis have come into existence like skiagraphs. Yet the unfortunate general practitioner in the midst of unceasing toil and with very scanty leisure is expected by his patients to be well versed in all and to apply each at a moment's notice. And he is not only expected to apply his knowledge but he must know and be able to give reasons for his use of this or that method and must explain away the erroneous notions which have been obtained from an ill-informed writers in the lay press. Still he obtains his facts and applies them in a very satisfactory manner for the most part and all credit is due to him for his success.

Sir William Church in his recent address on Medical Legislation and Education delivered at the opening of the Winter Session of the Post-graduate College, West London Hospital, very properly points out that the earlier attempts to foster post-graduate study failed mainly because they took the form of didactic lectures, and did not embrace hospital practice. The modern system is much more satisfactory, as it aims at the foundation of a complete medical school in which the students are qualified medical men so that there may be no jealousy between them and the junior students, as might very possibly happen in the medical schools attached to our large general hospitals.

